# Bacteriocins in Cancer Treatment: Mechanisms and Clinical Potentials

**DOI:** 10.3390/biom14070831

**Published:** 2024-07-10

**Authors:** Yiwen Wang, Yue Wang, Tao Sun, Junnan Xu

**Affiliations:** 1 Department of Breast Medicine 1, Cancer Hospital of China Medical University, Liaoning Cancer Hospital, Shenyang 110042, China; 2022122121@cmu.edu.cn (Y.W.); 2023122284@cmu.edu.cn (Y.W.); 2 Department of Pharmacology, Cancer Hospital of China Medical University, Liaoning Cancer Hospital, Shenyang 110042, China; 3 Department of Breast Medicine, Cancer Hospital of Dalian University of Technology, Liaoning Cancer Hospital, Shenyang 110042, China

**Keywords:** antimicrobial peptide, bacteriocin, cancer, mechanism, clinical application

## Abstract

Cancer poses a severe threat to human health. Although conventional chemotherapy remains a cornerstone of cancer treatment, its significant side effects and the growing issue of drug resistance necessitate the urgent search for more efficient and less toxic anticancer drugs. In recent years, bacteriocins, antimicrobial peptides of microbial origin, have garnered significant attention due to their targeted antitumor activity. This unique activity is mainly attributed to their cationic and amphiphilic nature, which enables bacteriocins to specifically kill tumor cells without harming normal cells. When involving non-membrane-disrupting mechanisms, such as apoptosis induction, cell cycle blockade, and metastasis inhibition, the core mechanism of action is achieved by disrupting cell membranes, which endows bacteriocins with low drug resistance and high selectivity. However, the susceptibility of bacteriocins to hydrolysis and hemolysis in vivo limits their clinical application. To overcome these challenges, structural optimization of bacteriocins or their combination with nanotechnology is proposed for future development. This review aims to study the mechanism of action and current research status of bacteriocins as anticancer treatments, thus providing new insights for their clinical development and application.

## 1. Introduction

Cancer is the leading cause of death worldwide. According to the latest statistics from the International Agency for Research on Cancer (IARC), in 2022, there were an estimated 20 million new cancer cases and 9.7 million cancer deaths [1]. Current available cancer treatments include chemotherapy, surgery, and radiation therapy, with chemotherapy being the predominant treatment option [2]. However, conventional chemotherapeutic drugs exhibit poor specificity for cancer cells, causing damage to normal cells and leading to serious side effects. Moreover, due to various factors, such as increased drug efflux or drug inactivation, altered or non-existent targets of drug action, and DNA damage repair [3], cancer cells become resistant to existing chemotherapeutic drugs, significantly reducing the efficacy of chemotherapeutic drugs. Therefore, there is an urgent need to discover new anticancer drugs with fewer side effects and a lower likelihood of resistance development.

Antimicrobial peptides (AMPs), also known as host defense peptides, are important components of the host’s innate immune system and are widely found across various organisms, including animals, plants, and microorganisms [4]. Many studies have reported the anticancer activity of AMPs isolated from different sources [5,6,7]. Among them are AMPs isolated from bacteria, also called bacteriocins, whose anticancer activity has been demonstrated in many cancer cell lines, such as lung, breast, liver, stomach, and colon cancer cells [8,9,10,11,12]. In fact, as early as the 1970s and early 1980s, bacteriocins received significant attention for their unique targeted antitumor activity [13]. However, the subsequent enthusiasm for the antimicrobial applications of bacteriocins overshadowed research on their anticancer potential [13]. Recently, with the popularization of peptide therapy and bacteriotherapy, interest in exploring bacteriocins as novel anticancer drugs has been rekindled [14].

Bacteriocins are derived from a wide range of sources. In addition to bacteria found in water, soil, and food, the human gastrointestinal microbiota is also an important source [15]. Almost all bacteria can produce at least one bacteriocin [16], which exhibits either a narrow or broad spectrum of bactericidal or bacteriostatic activity [17]. Like eukaryotic AMPs, bacteriocins are mostly cationic and amphiphilic in nature [18]. These cationic peptides selectively interact with cancer cells, which have negatively charged surfaces, forming transmembrane channels that disrupt cell membrane integrity, thereby killing cancer cells while being virtually harmless to normal cells [18]. In addition to membrane disruption, bacteriocins can also inhibit tumor development through mechanisms such as inducing apoptosis, blocking the cell cycle, and inhibiting cancer cell metastasis [17,19].

Bacteriocins hold great promise in cancer treatment. However, their clinical development is hindered by several unavoidable defects. To improve the stability and bioavailability of bacteriocins in vivo and reduce their toxicity to normal cells, structural optimization of bacteriocins or the use of nanocarriers as delivery systems is required. This review briefly describes the characteristics and classification of bacteriocins, examines their main mechanisms of action in malignant tumors, discusses their potential for cancer treatment alone or in combination with other drugs, and elucidates the challenges and future research directions for their clinical translation. Our goal is to deepen the understanding of bacteriocins and to provide new insights into the application of bacteriocins in cancer treatment.

## 2. Bacteriocin

Bacteriocins are multifunctional AMPs synthesized by different bacteria via ribosomes, usually small peptides consisting of 20 to 60 amino acid residues, but a few are proteins [20]. Most known bacteriocins are cationic, probably due to excessive lysine or arginine residues in their amino acid sequences [18]. In addition, amphiphilicity and hydrophobicity are important properties of bacteriocins, and these properties of bacteriocins are key to their activity. Bacteriocins have the following four main secondary structures: α-helix, β-sheet, extended helices, and loop structures. Among them, the α-helix structure is the predominant and most studied secondary structure. This structure can be described as an almost ideal amphiphilic structure with a positively charged hydrophilic region on one side of the helical axis and a hydrophobic region on the other [5]. Notably, bacteriocins are usually unstructured in aqueous solution and do not form active structures until they interact with target cell membranes [18].

Bacteriocins were once thought to inhibit only closely related species [21]. However, this claim became untenable with the discovery of broad-spectrum bacteriocins [22,23]. Bacteriocins have multiple mechanisms to kill bacteria, including inhibition of peptidoglycan biosynthesis, targeting specific receptors, and membrane disruption [17]. Lipid II, a key intermediate in peptidoglycan biosynthesis, can bind directly to lantibiotics such as nisin, which effectively blocks peptidoglycan formation [24]. Meanwhile, lipid II is also a receptor for many bacteriocins. For example, Lacticin 3147 utilizes lipid II as a docking molecule, and upon binding, they form pores in the bacterial membrane, leading to the spillover of internal bacterial components and ultimately triggering bacterial death [25]. In addition to lipid II, the mannose phosphotransferase system (Man-PTS) is also an important receptor for bacteriocins, which has been widely reported in the antimicrobial mechanism of pediocin-like bacteriocins [17]. It should be noted that among the various antimicrobial mechanisms of bacteriocins, the membrane disruption mechanism dominates. This mechanism also kills bacteria by forming pores in the bacterial membrane, which is not achieved by binding to a specific receptor but by electrostatic interactions. Specifically, since anionic molecules such as phosphatidylserine (PS), lipopolysaccharide (Gram-negative bacteria), and lipoteichoic acid (Gram-positive bacteria) are present in large quantities on the surface of bacteria, they are thus negatively charged. Cationic bacteriocins bind to the bacterial surface through electrostatic interactions, leading to pore formation and bacterial death [26,27]. Interestingly, bacteriocin-producing bacteria have immune proteins in their cell membranes, so they do not kill themselves [19]. Bacteriocins exhibit more potent antibacterial effects than eukaryotic AMPs, often showing efficacy at picomolar to nanomolar concentrations [17]. Unlike antibiotics, bacteriocins are primary metabolites and therefore more readily available by biosynthetic means. Furthermore, bacteriocins are colorless, odorless, and tasteless and generally exhibit high thermal stability and a wide pH range, all of which provide favorable conditions for their application [17,28]. Of course, the most significant advantage of bacteriocins over antibiotics is their low propensity to induce resistance [29]. It has been nearly a century since the discovery of bacteriocins, but no article has reported the emergence of drug resistance to bacteriocins. This may be because bacteriocins have a powerful and rapid bactericidal ability, making bacteria less likely to develop resistance even at lower concentrations [28]. On the other hand, bacteriocins can be hydrolyzed by proteases and do not persist for long periods, significantly reducing the possibility of bacteriocin resistance [28]. This advantage makes bacteriocins highly likely to become an antibiotic alternative, especially when antibiotic resistance is a growing problem [30]. In addition, bacteriocins are widely used in the food industry. Currently, nisin and pediocin PA-1 have been approved by the Food and Drug Administration (FDA) and commercialized on a large scale as food preservatives [31].

### 2.1. Classification of Bacteriocins

**Figure 1:**
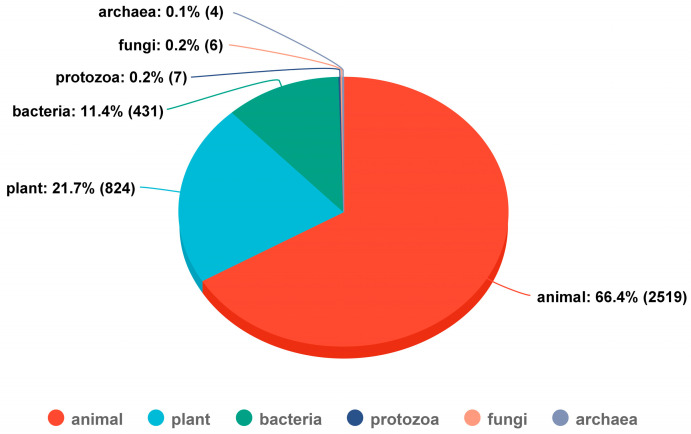
Various sources of antimicrobial peptides and the quantity and proportion of each source. Data were obtained from the DRAMP database. DRAMP, Data Repository of Antimicrobial Peptides.

Bacteriocins were first discovered by Gratia in 1925 when he observed that a strain of *Escherichia coli* produced a toxic substance that could have an antagonistic effect on neighboring strains of *Escherichia coli* [17]. He named this substance Colicin V. With the continuous development of isolation, purification and identification techniques, an increasing number of bacteriocins have been discovered, with 431 bacteriocins currently cataloged in the Data Repository of Antimicrobial Peptides (DRAMP) database [32] (Figure 1). Bacteriocins vary widely in size, characterization, antimicrobial spectrum, amino acid sequence, and spatial structure, necessitating a classification standard to facilitate research. Kaur et al. [14] improved upon their predecessors [20,21,33,34] by classifying bacteriocins into three categories according to their molecular weight and biochemical properties.

#### 2.1.1. Class I Bacteriocins

Class I bacteriocins, also known as Lantibiotics, are a class of highly post-translationally modified peptides that are thermally stable and have small molecular weights (<5 kDa). Lantibiotics are characterized by some uncommon amino acids in the sequence, including lanthionine, methyllanthionine, dehydroalanine, and dehydrobutyrine, which can form intramolecular thioether rings. Lantibiotics are classified into two subtypes based on their chemical properties and antimicrobial mechanisms. Type A Lantibiotics (2–4 kDa), such as nisin and lacticin 481, are positively charged, screw-shaped peptides with high conformational freedom. They act as bactericides via a membrane disruption mechanism. Type B Lantibiotics (2 to 3 kDa) are uncharged or negatively charged. They are globular peptides with a stable structure, and their antimicrobial activity is associated with the inhibition of peptidoglycan biosynthesis and the interference of cellular enzymatic reactions [35,36]. The cinnamycin secreted by *Streptomyces cinnamoneus* is an example of this type.

#### 2.1.2. Class II Bacteriocins

Class II bacteriocins are small peptides (0.7 to 10 kDa) that are not post-translationally modified. These bacteriocins are thermally stable and active over a wide pH range. They are further classified into three subclasses. Subclass IIa bacteriocins, such as pediocin PA-1 and enterocin A, contain a highly conserved YGNGVXC sequence at the N-terminus, and all of them are highly inhibitory to *Listeria monocytogenes*. Subclass IIb bacteriocins are two-component bacteriocins that require the synergistic action of two peptides to exert their antimicrobial effects. Plantaricin A and plantaricin P1053 are grouped as subclass IIb bacteriocins. Subclass IIc bacteriocins are a class of cyclic bacteriocins formed by peptide bonding, such as enterocin AS48 and circularin A.

#### 2.1.3. Class III Bacteriocins

Class III bacteriocins are proteins with high molecular weights (>10 kDa) that are thermally unstable. Representative class III bacteriocins include colicins produced by *Escherichia coli* and pyocin S2 produced by *Pseudomonas aeruginosa* 42A.

## 3. Anticancer Mechanisms of Bacteriocins

**Figure 2:**
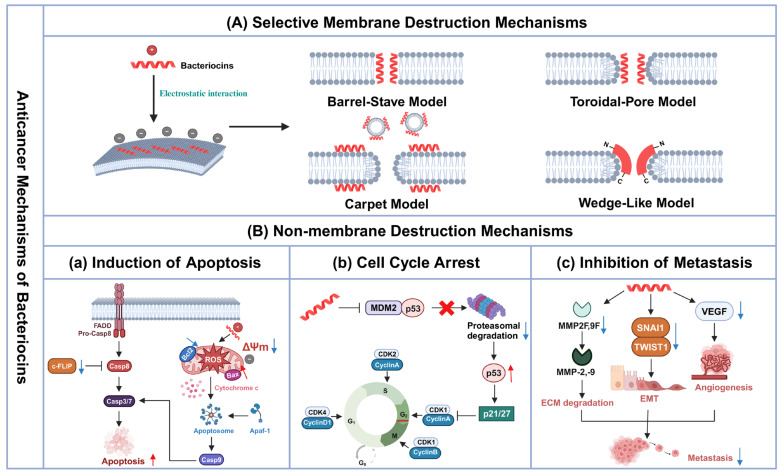
Schematic diagram of the anticancer mechanism of bacteriocins. (**A**) In the membrane disruption mechanism, cationic bacteriocins increase membrane permeability and efflux of intracellular contents through electrostatic interactions with negatively charged cancer cell membranes, ultimately leading to cell death. There are four main membrane pore formation models: the barrel-stave, carpet, toroidal-pore, and wedge-like models. (**B**) After entering the cell membrane, bacteriocins can act on intracellular structures or molecules to initiate non-membrane disruption mechanisms. (**a**) Induction of apoptosis: On the one hand, due to the negative charge of the mitochondrial membrane, bacteriocins can directly induce mitochondrial membrane disruption, leading to a decrease in mitochondrial membrane potential. In addition, some bacteriocins can increase the permeability of the mitochondrial membrane by upregulating the ratio of Bax to Bcl-2 and a large amount of ROS production. Eventually, cytochrome C is released into the cytoplasm and binds to Apaf-1 to form apoptosomes, which activates caspase-9 and initiates the caspase-dependent apoptotic program. On the other hand, bacteriocins can promote apoptosis through the death receptor pathway by inhibiting the expression of the caspase-8 inhibitor c-FLIP. (**b**) Cell cycle arrest: Bacteriocins cause cell cycle arrest by precisely regulating the expression of different cyclin and CDKs. Some bacteriocins block the binding of MDM2 to p53, thereby diminishing p53 degradation via the proteasome pathway and significantly increasing p53’s intracellular concentration. This increase in p53 enhances the transcription of CDK inhibitors p21 and p27, consequently leading to a marked reduction in CDK2 and cyclin A levels. This series of events culminates in cell cycle arrest during the G2/M phase. (**c**) Inhibition of metastasis: Bacteriocins inhibit ECM degradation, EMT, and angiogenesis by downregulating the expression of genes or proteins such as MMPs, SNAI1, TWIST1, and VEGF, ultimately inhibiting tumor cell metastasis. Red arrows indicate the up-regulation of gene or protein expression levels, while blue arrows indicate the down-regulation of gene or protein expression levels. Black arrows indicate promotional effects, while black horizontal bars indicate inhibitory effects. FADD, FAS-associated protein with death domain; Casp, caspase; Bcl-2, B-cell lymphoma-2; Bax, Bcl-2-associated X; ΔΨm, mitochondrial membrane potential; ROS, reactive oxygen species; Apaf-1, apoptotic protease activating factor-1; c-FLIP, cellular FLICE-like inhibitory protein; MDM2, murine double minute 2; CDKs, cyclin-dependent kinases; ECM, extracellular matrix; EMT, epithelial–mesenchymal transition; MMPs, matrix metalloproteinases; VEGF, vascular endothelial growth factor. Created with BioRender.com. (accessed on 2 July 2024).

In addition to antimicrobial activity, many bacteriocins exhibit significant targeted antitumor activity. The anticancer mechanisms of bacteriocins are not fully elucidated, but the widely accepted mechanisms include selective membrane disruption and non-membrane disruption (Figure 2). Notably, these two mechanisms are not completely independent, with bacteriocins potentially interacting with the cancer cell membrane first, entering the cell, and then acting on various intracellular targets to induce cancer cell death.

### 3.1. Selective Membrane Destruction Mechanisms

The selective membrane disruption mechanism, also known as the extracellular mechanism, is the main anticancer mechanism of bacteriocins. However, the primary question is how can bacteriocins selectively kill cancer cells without affecting normal cells? Similar to their antimicrobial mechanisms, the specificity of cancer cell membranes underlies the anticancer activity of bacteriocins. In normal cells, the outer surface of the cell membrane is enriched with zwitterionic phospholipids, such as phosphatidylcholine and sphingomyelin, making it generally neutral [37]. However, the outer surface of the cell membrane in cancer cells has a net negative charge due to the overexpression of anionic molecules such as phosphatidylserine [37], O-glycosylated mucins [38], sialylated gangliosides [39], and heparin sulfates [40], which facilitate electrostatic interactions between cancer cells and positively charged bacteriocins. In addition, cancer cell membranes are more fluid and contain more microvilli than normal cell membranes [41,42], both of which contribute to the metastatic ability of cancer cells [43,44]. However, the increased fluidity of cancer cell membranes destabilizes the membrane, enhancing bacteriocins’ cleavage ability [45]. Moreover, microvilli of cancer cells can significantly increase the total surface area of cancer cells, allowing more bacteriocins to bind to the cancer cell membranes [46], thus considerably enhancing the anticancer effect of bacteriocins.

Researchers have summarized four models based on the interaction of bacteriocins with cell membranes, including the barrel-stave model, the carpet model, the toroidal-pore model, and the wedge-like model [47,48]. The exact model through which bacteriocins act depends mainly on membrane composition, peptide structure, peptide concentration, and lipid membrane properties [45].

#### 3.1.1. Barrel-Stave Model

In the barrel-stave model, bacteriocins, usually with amphiphilic α-helix structures, such as acidocin J1132 and acidocin J1229 [49,50], change conformation after binding to the target cell membrane and insert vertically into the phospholipid bilayer through the hydrophobic region in the α-helix structure. A single bacteriocin is equivalent to a stave in a barrel. Upon aggregation, the hydrophilic side can form a “barrel”-shaped transmembrane channel, which causes the efflux of intracellular substances; disruption of the ionic gradient and transmembrane potential; and, ultimately, cell death [51].

#### 3.1.2. Carpet Model

Unlike the barrel-stave model, the carpet model has no structural requirements for bacteriocins and does not form transmembrane channels. In the carpet model, bacteriocins bind to the cell membrane through electrostatic interactions and then arrange parallel to the cell membrane surface like a “carpet”. When a critical concentration of bacteriocins is reached, the hydrophilic amino acids of bacteriocins interact with the polar heads of phospholipids to form micelles, which penetrate the cell membrane and ultimately lead to disintegration [52]. Plantaricin 149 derived from *Lactobacillus plantarum* NRIC 149 exerts its membrane-disrupting effect through this model, with a critical concentration of approximately 2.5 μM [53].

#### 3.1.3. Toroidal-Pore Model

The toroidal-pore model combines features of the above two models [45]. In this model, bacteriocins align parallel to the cell membrane surface after binding to the cell membrane through electrostatic interactions. Once the bacteriocin reaches a certain concentration, interactions between the hydrophilic amino acids of the bacteriocin and the polar heads of the phospholipids occur, causing the lipid monolayer to undergo continuous inward bending for stable curvature and to form transmembrane toroidal pores [51]. The hydrophilic groups of bacteriocins and lipids always face together toward the center of the pore. Most bacteriocins, including lacticin Q, colicin E1, and colicin U [54,55,56], exert membrane-disrupting effects through this model.

#### 3.1.4. Wedge-like Model

The wedge-like model, discovered in the membrane disruption mechanism of nisin [57], is similar to the toroidal-pore model. Nisin binds to the target cell membrane and, upon reaching a threshold concentration, the bound anionic phospholipid and nisin’s structural domain co-insert into the membrane [58]. Since hinges in the nisin molecule may allow partial bending of the C-terminus, the transmembrane pores are wedge-like [58]. Recently, Zhu et al. found that pediocin PA-1 also acts through this model [59]. However, studies have shown that wedge-like and toroidal pores may be unstable, with hydrophobic forces driving the phospholipid bilayer to rearrange itself into its original structure [47,51]. Still, bacteriocins can enter the cell through the pore and exert non-membrane disruption mechanisms to kill the cell.

### 3.2. Non-Membrane Destruction Mechanisms

The non-membrane disruption mechanisms of bacteriocins, also called intracellular mechanisms, include the induction of apoptosis, cell cycle arrest, and inhibition of metastasis.

#### 3.2.1. Induction of Apoptosis

The mitochondrial membrane surface of cancer cells carries a net negative charge due to the enrichment of anionic lipids, such as cardiolipin [60]. Thus, some cationic bacteriocins, such as nisin and Microcin E492 [61,62], can target mitochondria and disrupt the integrity of the mitochondrial membrane, which leads to a decrease in the mitochondrial membrane potential and promotes the release of cytochrome c from the mitochondria into the cytoplasm. Cytochrome c binds to apoptotic protease activating factor-1 (Apaf-1) to form apoptosomes, which activate caspase-9. Subsequently, activated caspase-9 further activates downstream caspase-3, ultimately triggering the caspase-dependent apoptosis pathway. However, Punj et al. found that azurin does not act directly on mitochondria but rather by upregulating the ratio of Bax to Bcl-2 and promoting the release of reactive oxygen species (ROS) to increase the permeability of the mitochondrial membrane [63]. Ultimately, apoptosis is induced by activating caspase-7 but not caspase-3. In addition, Arunmanee et al. observed that the expression of the anti-apoptotic protein myeloid cell leukemia-1 (Mcl-1) and the caspase-8 inhibitor cellular FLICE-like inhibitory protein (c-FLIP) was significantly decreased, while the expression of Bax was considerably upregulated after colicin N was applied to cancer cells [8]. These results indicate that colicin N activates the mitochondrial and death receptor pathways together to induce apoptosis.

#### 3.2.2. Cell Cycle Arrest

Many bacteriocins can inhibit cancer cell proliferation by blocking various cell cycle phases, including the G0, G1, S, G2 and M phases. As a fundamental process of cellular life activities, the cell cycle is precisely regulated by cyclins and cyclin-dependent kinases (CDKs). Cyclin D1 activates many CDKs, mainly CDK4, which drives the cell cycle, transforming G1 to S phase [64]. Hosseini et al. found that nisin inhibited the expression of cyclin D1, leading to the cell cycle being blocked in the G1 phase and significantly inhibiting the proliferation of cancer cells [65]. p53 is an important tumor suppressor, and one of its core functions is to block the cell cycle [66]. Azurin and its derivative p28 can form a complex with p53, which blocks the binding of murine double minute 2 (MDM2) to p53, thereby reducing the degradation of p53 through the proteasome pathway and significantly elevating the intracellular concentration of p53. An increase in the level of p53 upregulated the expression of the CDK inhibitors p21 and p27, which further led to a significant decrease in the intracellular levels of CDK2 and cyclin A. These changes induced cell cycle arrest in the G2/M phase, ultimately inhibiting the proliferation of various cancer cells, such as breast cancer and melanoma cells [67,68].

#### 3.2.3. Inhibition of Metastasis

Nisin can inhibit various factors associated with cancer metastasis, including extracellular matrix (ECM) degradation, epithelial-mesenchymal transition (EMT), and tumor angiogenesis. First, nisin downregulates the expression of the MMP2F and MMP9F genes, significantly affecting matrix metalloproteinase (MMP) activity [69]. MMPs, especially MMP-2 and MMP-9, play essential roles in cancer metastasis by degrading key components of the ECM, thereby creating pathways for cancer cells to invade and metastasize [70]. However, nisin effectively impedes this process, reducing the risk of cancer cells breaking through the ECM barrier and spreading to surrounding tissues. Second, Balcik-Ercin et al. reported that treatment of the hepatocellular carcinoma cell line HuH-7 with nisin significantly suppressed the expression of SNAI1 and TWIST1, which are essential regulators of the EMT process [12]. Therefore, the downregulation of their expression inhibited EMT onset, thereby reducing cancer cells’ metastatic potential. Finally, nisin was also found to significantly reduce the level of vascular endothelial growth factor (VEGF), thereby inhibiting tumor angiogenesis and ultimately preventing cancer cells from entering the circulation through blood vessels to achieve metastasis [71].

## 4. Clinical Potential of Bacteriocins in Cancer Treatment

**Figure 3:**
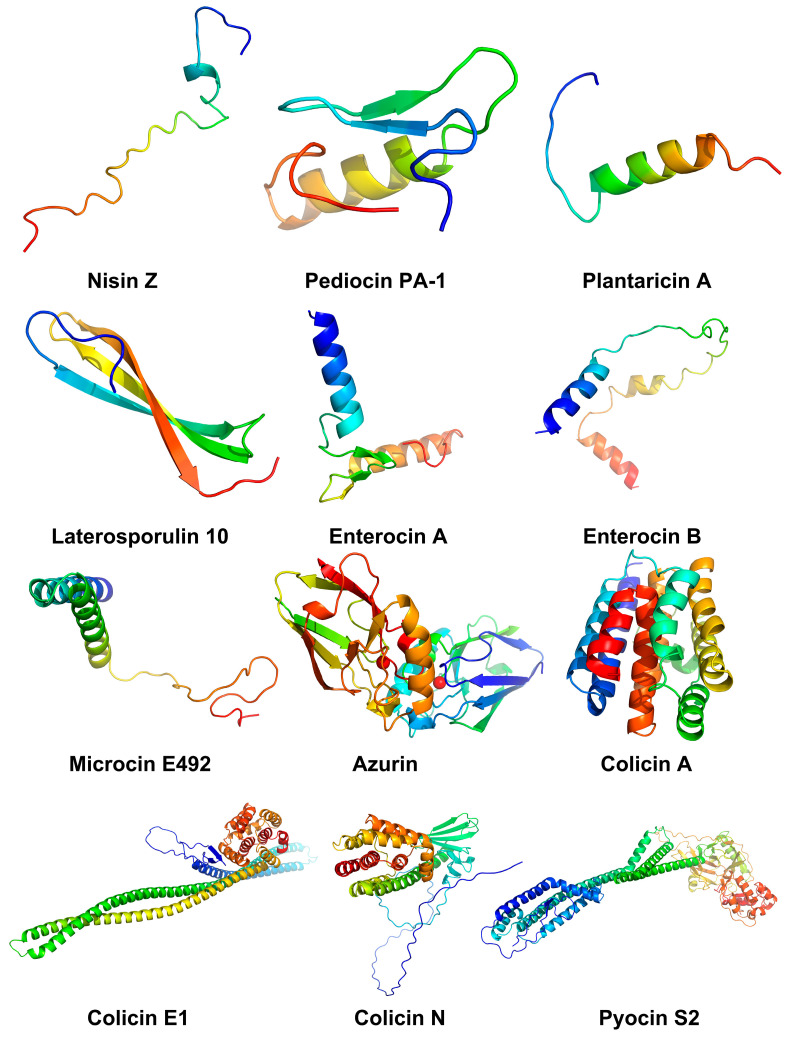
Structure of bacteriocins with antitumor activity. Procedure: (1) amino acid sequences of bacteriocins were downloaded from PDB and UniProt databases; (2) homology modeling server SWISS-MODEL was used to predict the tertiary structure of bacteriocins; (3) PyMOL 2.6.0 software was used to visualize these structure. PDB, Protein Data Bank; UniProt, Universal Protein.

Although the current clinical application of bacteriocins mainly focuses on the treatment of pathogenic bacterial infections, an increasing number of in vitro and in vivo studies have shown that bacteriocins have significant antitumor activity and potential as a new type of anticancer drug (Table 1, Figure 3). Conventional anticancer drugs suffer from high toxicity and side effects and susceptibility to drug resistance. However, bacteriocins selectively destroy cancer cells without harming neighboring normal cells. Moreover, this mechanism allows bacteriocins to enter cancer cells and exert rapid tumor-killing effects without having to interact with a specific receptor, decreasing susceptibility to developing drug resistance. Given these advantages, bacteriocins may have broad application prospects in cancer treatment alone or in combination with anticancer drugs.

### 4.1. Bacteriocins Alone for Cancer Treatment in Clinical Study

Current research on bacteriocins in cancer treatment has focused mainly on the non-clinical phase (in vitro and in vivo studies), while there is a relative scarcity of clinical studies. To date, only two bacteriocins, azurin-p28 and nisin ZP, have successfully entered the human phase I/II clinical trial phase to evaluate their safety, tolerability, pharmacokinetics, and preliminary efficacy as novel antitumor therapies (Table 2).

p28 (NSC745104) is a cell-penetrating peptide derived from azurin (containing amino acids 50–77 of azurin) [104]. Two clinical trials were conducted on p28, and both were completed. Warso et al. conducted the first phase I clinical trial of p28 in 2009 (NCT00914914), enrolling 15 patients with p53-positive advanced solid tumors [105]. These patients were divided into five groups, with patients in the first group receiving intravenous p28 three times per week for four weeks, starting at the lowest dose (0.83 mg/kg) and monitoring for two weeks. If no adverse effects were observed, patients continued to receive increasing doses of p28 (1.66, 2.5, 3.33, and 4.16 mg/kg) sequentially in the same manner. At the same time, the next group of patients was added, starting treatment at the dose level at which the previous group had progressed. The results showed that patients tolerated p28 well, even at the highest dose, with no adverse effects. Moreover, p28 showed good efficacy, with seven patients in stable condition (7–61 weeks), three in partial remission (44–125 weeks), and one in complete remission (139 weeks). Three patients were still alive as of the last follow-up in December 2012. Another phase I clinical trial of p28 (NCT01975116) was initiated by the Pediatric Brain Tumor Consortium, including 18 pediatric patients aged 3 to 21 years with progressive, recurrent, or refractory central nervous system (CNS) tumors [106]. The dosing regimen was similar to that used in the first study, with intravenous p28 administered three times per week for four weeks and then monitored for two weeks. The starting dose of p28 was 4.16 mg/kg, which was repeated every six weeks for up to ten courses without any observed disease progression or serious adverse effects. The results showed that, except for six patients who discontinued treatment due to disease progression, p28 was safe and well tolerated in the remaining patients and produced responses in some of them. CNS tumors are often difficult to treat and have a high mortality rate due to the presence of the blood–brain barrier (BBB), which makes it difficult for drugs to reach the tumor site. However, p28, a cell-penetrating peptide consisting of 28 amino acid residues, can cross the BBB and enter brain cells, opening up new possibilities for treating CNS tumors.

Nisin, produced by *Lactococcus lactis*, is a broad-spectrum bacteriocin with two variants: Nisin A and Nisin Z [107]. The only difference between them is the 27th amino acid, where Nisin A contains histidine (His) and Nisin Z contains asparagine (Asn). Nisin ZP, an ultrapure concentrate of Nisin Z, is currently being tested in a clinical trial (NCT06097468) designed to enroll 40 adult patients with non-metastatic oral squamous cell carcinoma (OSCC). Participants who meet the inclusion criteria will receive Nisin ZP orally once daily for two weeks prior to resection surgery for OSCC. Nisin ZP will be temporarily discontinued during surgery and will continue to be administered once daily for six months after surgery (this process will be synchronized with conventional and adjuvant therapies). The starting dose is set at 20,000 mg daily, but dose adjustments will be made based on patient toxicity response or tolerability. Upon entry into phase II, the dosing regimen will be similar to that in phase I. However, dose levels will be adjusted based on efficacy and safety data from phase I, again considering patient toxicity or tolerability.

### 4.2. Bacteriocins Combined with Anticancer Drugs for Cancer Treatment

In addition to being used alone, bacteriocins can be combined with conventional anticancer drugs to exert antitumor effects. Conventional anticancer drugs usually need to bind to a specific receptor to enter the cell to be effective. However, when combined with bacteriocins, bacteriocins can disrupt the membrane structure of tumor cells, facilitating the entry of anticancer drugs, improving drug utilization, reducing administration frequency, and helping to prevent or delay drug resistance and severe side effects.

Preet et al. investigated the effect of nisin in combination with doxorubicin (DOX) on 7,12-dimethylbenz (a) anthracene (DMBA)-induced skin cancer using a mouse model [108]. The mice were randomly divided into a control group (tumor-untreated group) and a test group, where the test group was subdivided into a DOX-alone group, a nisin-alone group, and a nisin+DOX combination treatment group. After four weeks of treatment, compared with untreated control mice, mice treated with DOX or nisin alone showed a 39.69% and 10.07% reduction in the mean tumor volume and a 51.3% and 14.18% reduction in the mean tumor burden, respectively. However, these data were even more dramatically improved in the nisin+DOX combination treatment group, which significantly reduced the tumor volume by 58.16%, and moreover, the tumor burden by up to 66.82%. Furthermore, they also observed that nisin+DOX combination treatment induced a significant increase in the percentage of apoptotic cells in the tumor tissues of mice. Another example of the use of nisin in combination with anticancer drugs came from a study by Mohammadi et al., who reported that combination therapy with nisin and rituximab was more effective at increasing cytotoxicity and apoptosis in the human Burkitt lymphoma cell lines Raji and Daudi than treatment with nisin or rituximab alone at their respective IC_50_ concentrations [109].

In addition, azurin has been found to have synergistic antitumor effects with various anticancer drugs. In vitro studies have shown that when YD-9 (OSCC) cells and MG-63 (human osteosarcoma) cells were treated with 5-fluorouracil (5-FU) or etoposide alone, these anticancer drugs inhibited the growth of only approximately 30% of the cells, even at a concentration of 1 mM [95]. However, when 5-FU or etoposide was combined with azurin, a concentration of only 10 μM was sufficient to achieve 80–90% cell growth inhibition, suggesting that the combination regimen of azurin with 5-FU or etoposide can significantly improve the sensitivity of OSCC and human osteosarcoma to anticancer drugs.

The above studies demonstrated the significant cumulative effect of combining bacteriocins with anticancer drugs in tumor therapy. Bacteriocins are expected to be a powerful complement to existing anticancer drugs, opening up new possibilities for cancer treatment.

## 5. Challenges and Ways Forward

Bacteriocins have shown great promise in cancer treatment due to their targeted antitumor activity and low susceptibility to drug resistance. However, many limitations still need to be overcome before being formally put into clinical use. Firstly, bacteriocins suffer from poor in vivo stability, short half-lives, and low bioavailability due to susceptibility to protease degradation in serum and gastrointestinal environments, as well as rapid hepatic and renal clearance. Secondly, some bacteriocins are hemolytic and toxic to normal cells. In addition, the efficacy of bacteriocins remains relatively low compared to that of marketed anticancer drugs. Finally, the traditional methods used to produce and purify bacteriocins are complex and have low yields. Bacteriocins purified from culture supernatants through multiple steps, such as ammonium sulphate precipitation, ion-exchange chromatography, and hydrophobic interaction chromatography, are typically found at concentrations less than 1 mg per liter of culture [14]. Given the short sequence of class I bacteriocins, the chemical synthesis of peptides can also be considered. However, due to their high degree of complexity and post-translational modifications, they are costly and challenging to adapt to the needs of large-scale commercialization [28]. Therefore, the way in which to achieve large-scale production and purification of bacteriocins is also one of the problems that must be solved in practical applications.

To overcome the poor pharmacodynamic and pharmacokinetic properties of bacteriocins, three ideas can be considered. One is to optimize the structure of bacteriocins. D-amino acid substitution is the most commonly used modification because D-amino acids are less susceptible to protease hydrolysis in vivo [110,111]. Bengtsson et al. replaced all the L-amino acids in the sequence of Plantaricin NC8 αβ with D-amino acids [112]. After 16 h of trypsin treatment, the D-enantiomer exhibited greater stability than the L-enantiomer, and its antimicrobial activity was unaffected. Partial D-amino acid substitutions, such as lactococcin G analogs (substituting N- and C-terminal residues with D-amino acids), also enhance stability against proteases without compromising antimicrobial activity [113]. Interestingly, many studies have shown that cyclic bacteriocins, such as AS-48, are very stable in vivo with only minor hydrolysis. Comparison with their linear derivatives confirmed that the cyclic structure is the key to stability [114]. This finding suggested that cyclization may be a promising method for improving bacteriocin stability.

The second strategy is based on genetic engineering technology. Researchers have used this technique to design fusion proteins and introduce affinity tags, thus offering the possibility of large-scale production and purification of bacteriocins [13]. What is more, genetic engineering technology also has great potential for the modification of bacteriocins. On the one hand, genetic engineering techniques have been utilized to induce site-directed alterations in the protease recognition site of bacteriocins to produce protease-resistant variants, thereby improving their stability in vivo [115]. On the other hand, recombination of the genes of two or more bacteriocins to form a new peptide to enhance the therapeutic potential of bacteriocins, such as the combination of enterocin-A and enterocin-B and the enterocin A-colicin E1 fusion peptide, has been widely reported [90,116].

The final way is to utilize nanocarriers, which have many unique properties, such as long in vivo circulation, controlled drug release, and prevention of drug degradation [5], as drug delivery systems. In addition to serving as a means of overcoming the instability of bacteriocins, the combination of modern nanotechnology with bacteriocins can also improve target cell delivery, thus reducing the toxicity of free bacteriocins to non-target cells or organs, which will help promote the clinical application of bacteriocins [117]. β-Cyclodextrin-based nanosponges (β-CD-NSs) are excellent nanocarriers that not only possess the advantages of nontoxicity, biocompatibility, and biodegradability but also interact with biological tissues through their tunable functional groups, which provide the basis for targeted drug delivery [118]. It has been reported that the complexes formed by nisin Z upon binding to β-CD-NS showed significant activity against the colon cancer cell line HT-29 and the breast cancer cell line MCF-7 [118]. In addition, these complexes could protect nisin Z from degradation by pepsin and showed good sustained release effects, which were favorable for reducing the administered dose of nisin and decreasing potentially toxic side effects. In another study, Hashad et al. utilized ultrasonication technology and the “bio-click” method to successfully construct new nanovesicles (NSNE_DOX_-αHER2 IgG), which consisted of a DOX-loaded nisin shell nanoemulsion surface decorated with the cancer-targeting ligand αHER2 IgG [119]. These nanovesicles were verified to be stable in plasma by in vitro experiments, and because of their ability to precisely target HER2-positive cancer cells, their antitumor activity was significantly higher than that of nisin-shelled nanoemulsions (NSNE), DOX, or NSNE_DOX_ alone, with little effect on healthy cells.

## 6. Conclusions and Perspective

Due to the high toxicity and insufficient specificity and susceptibility to drug resistance of conventional anticancer drugs, the development and use of novel drugs to provide more precise and effective cancer treatments have become a matter of urgency. Many in vivo and in vitro studies have reported that bacteriocins can produce significant inhibitory effects on different cancer cell lines at nanomolar or micromolar concentrations in recent years. The cationic, amphiphilic, and hydrophobic nature of bacteriocins is the key to their antitumor ability. The negatively charged cancer cell membrane is the main target of bacteriocins, which bind to the cancer cell membrane through electrostatic interactions, forming transmembrane pores and leading to the death of cancer cells. Compared with traditional anticancer drugs, bacteriocins, due to their unique structural properties and anticancer mechanism, can not only precisely target cancer cells without harming normal cells but also have meager drug resistance, making them an ideal cancer treatment. Additionally, bacteriocins can be used as adjuvants to traditional anticancer drugs, helping reduce toxic side effects, delay the development of drug resistance, and significantly enhance the efficacy of both anticancer drugs and bacteriocins.

However, although bacteriocins show great potential in cancer treatment as an emerging therapeutic approach, they still face many challenges in clinical application. The major obstacles include poor in vivo stability (susceptibility to hydrolysis by proteases), poor pharmacokinetic properties (short half-life and low bioavailability), potential risk of hemolysis (a few bacteriocins), possible toxicity to normal cells, and high production costs. Future studies could consider combining bacteriocins with genetic engineering techniques and modern nanotechnology or optimizing their structures using D-amino acid substitutions and cyclization to overcome these challenges. Moreover, despite encouraging preclinical findings, the translation of bacteriocins into clinical practice remains limited, with only azurin and nisin Z advancing to clinical trials. Therefore, designing and conducting more clinical trials to thoroughly evaluate the anticancer efficacy and safety profiles of bacteriocins in cancer therapy will also be an important direction for future research.

## Figures and Tables

**Table 1:** Anticancer activity of bacteriocins on various cancer cells in vitro and in vivo studies.

Bacteriocin	Source	Uniprot ID/Sequence	Class	Size	Human Cancer Cell Line: IC_50_/Animal Model	Refs.
Nisin A	*Lactococcus lactis*	P13068/ ITSISLCTPGCKTGALMGCNMKTATCHCSIHVSK	I	3.5 kDa	HNSCC cell lines (UM-SCC-17B, UM-SCC-14A and HSC-3)/oral cancer mouse model [in vivo]: nisin (200 mg/kg) reduced tumor volume colon cancer cell lines (LS180: 80–400 IU/mL, (SW48, HT-29: 89.9 µM, Caco-2: 115.0 µM): 350–800 IU/mL and SW480: 1000 µg/mL) breast adenocarcinoma cell line (MCF-7: 105.46 µM) liver hepatocellular carcinoma cell line (HepG2: 112.25 µM, (HuH-7 and SNU182): ≈96 µM) T cell leukemia cell line (Jurkat: 225 µM) astrocytoma cell line (SW1088: 50 µg/mL) neuroblastoma cell line (IMR-32: >25 µM) cervical cancer cell line (HeLa: 11.5 µM) ovarian carcinoma cell lines (OVCAR-3: 14.6 µM and SK-OV-3: 22.9 µM)	[9,12,61,65,69,72,73,74,75,76]
Nisin Z	*Lactococcus lactis* SIK-83	P29559/ ITSISLCTPGCKTGALMGCNMKTATCNCSIHVSK	I	3.47 kDa	HNSCC cell lines (UM-SCC-17B, UM-SCC-14A and HSC-3)/oral cancer floor-of-mouth mouse model [in vivo]: nisin (400 and 800 mg/kg) reduced tumor volume lung carcinoma cell lines (A549: 132.4 µM and H1299: 137.3 µM) malignant melanoma cell line (A375: 188.5 µM)	[77,78,79,80]
Bovicin HC5	*Streptococcus bovis* HC5	N.A./ VGXRYASXPGXSWKYVXFXXVK	I	2.4 kDa	breast adenocarcinoma cell line (MCF-7: 279.39 µM) liver hepatocellular carcinoma cell line (HepG2: 289.30 µM)	[9]
Pep27anal2	*Streptococcus pneumoniae*	N.A./ MWKWFHNVLSWWWLLADKRPARDYNRK	I	3.3 kDa	acute myelogenous leukemia cell line (AML-2: 29 µM) acute promyelocytic leukemia cell line (HL-60: 20 µM) T cell leukemia cell line (Jurkat: 23 µM) gastric cancer cell line (SNU-601: 25 µM) breast cancer cell line (MCF-7: <10 µM)	[81]
Pediocin PA-1	*Pediococcus acidilactici* PAC 1.0	P29430	II	4.6 kDa	human lung carcinoma cell line (A-549: 1.66 µM) human colon adenocarcinoma cell line (DLD-1: 1.61 µM)	[82]
Pediocin K2a2–3	*Pediococcus acidilactici* K2a2–3	N.A./IYYGNGPTRGIHSRSPQGGIATTWVWNNGAMAHATGGHQ_____ (five residues missing)	II	4.6 kDa	colon adenocarcinoma cell line (HT-29): undialysed (1600 AU/mL): 55.0%; dialysed (800 AU/mL): 53.7% cervical carcinoma cell line (HeLa): undialysed (1600 AU/mL): 52.3%	[83]
Pediocin CP2	*Pediococcus acidilactici* CP2 MTCC5101	N.A.	II	4.6 kDa	hepatocarcinoma cell line (HepG2: <1 µg/mL) cervical adenocarcinoma cell line (HeLa: >1 µg/mL) mammary gland adenocarcinoma cell line (MCF-7: >1 µg/mL)	[84]
Plantaricin BM-1	*Lactobacillus plantarum* BM-1	N.A.	II	4.6 kDa	colorectal cancer cell lines (SW480: 757.9 µg/mL, Caco-2: 819.9 µg/mL, and HCT-116: 1578 µg/mL)	[10]
Plantaricin P1053	*Lactobacillus plantarum* strain PBS067	N.A.	II	1.05 kDa	colon cancer cell line (E705: >1 µg/mL)	[85]
Plantaricin A	*Lactobacillus plantarum* C11	P80214/KSSAYSLQMGATAIKQVKKLFKKWGW	II	2.99 kDa	T cell leukemia cell line (Jurkat): 25 µM at 37 °C B cell precursor acute lymphoblastic leukemia cell line (Reh: 5–20 µM)	[86,87]
Laterosporulin 10	*Brevibacillus* sp. *strain* SKDU10	N.A./ACVNQCPDAIDRFIVKDKGCHGVEKKYYKQVYVACMNGQHLYCRTEWGGPCQL	II	5.6 kDa	cervical cancer cell line (HeLa): <10 µM embryonic kidney cancer cell line (HEK293T): <10 µM fibro-sarcoma cell line (HT1080): <10 µM lung carcinoma (H1299): <10 µM breast cancer cell line (MCF-7): <10 µM	[88]
Enterocin A	*Enterococcus faecium*	C9BXY2/MKHLKILSIKETQLIYGGTTHSGKYYGNGVYCTKNKCTVDWAKATTCIAGMSIGGFLGGAIPGKC	II	7.24 kDa	colon cancer cell lines (HT-29: 50–120 µg/mL and Caco-2: 50–100 µg/mL) gastric cancer cell line (AGS: 50–100 µg/mL) cervical cancer cell line (HeLa: 25–100 µg/mL)	[89]
Enterocin B	*Enterococcus faecium*	A3FEQ6/TNVKELSTKEMKQIIGGENDHRMPNELNRPNNLSKGGAKCGAAIAGGLFGIPKGPLAWAAGLANVYS	II	7.2 kDa	cervical cancer cell line (HeLa: >25 µg/mL) colon cancer cell line (HT-29: >25 µg/mL) gastric cancer cell line (AGS: >25 µg/mL)	[90]
Microcin E492	*Klebsiella pneumoniae* RYC492	Q9Z4N4/GETDPNTQLLNDLGNNMAWGAALGAPGGLGSAALGAAGGALQTVGQGLIDHGPVNVPIPVLIGPSWNGSGSGYNSATSSSGSGS	II	7.89 kDa	cervix carcinoma cell line (HeLa: >680 AU/mL) T cell leukemia cell line (Jurkat: <680 AU/mL) a variant of the human Burkitt lymphoma B cell line (RJ2.2.5: >680 AU/mL) colorectal adenocarcinoma cell lines (HT-29: 60 µg/mL and SW620: <60 µg/mL)/zebrafish larvae SW620 xenograft model [in vivo]: significantly reduced the tumor size	[62,91]
Azurin	*Pseudomonas aeruginosa*	P00282	III	14 kDa	melanoma cell line (UISO-Mel-2: >800 µg/mL) human cancer xenotransplanted in nude mice [in vivo]: tumor volume was reduced by 59% in azurin-treated mice (0.5 mg) compared with controls breast cancer cell lines (MCF-7: 32 µM, MDD2: >60 µM, MDA-MB-157: 53 µM, and MDA-MB-231: >60 µM) nude mouse model with xenotransplanted MCF-7 cells [in vivo]: tumor volume was reduced by 85% in azurin-treated mice (1 mg) compared with controls osteosarcoma cell line (U2OS:114.54 mg/L) acute myeloid leukemia cell line (HL60: 2.5 µM) chronic myeloid leukemia cell line (K562: <2.5 µM) hepatocellular carcinoma cell line (HepG2: >8.0 µg/µL) colon carcinoma cell line (HCT-116: >10.0 µg/µL) oral squamous cell carcinoma cell line (YD-9: 200 µg/mL)	[63,67,92,93,94,95]
Colicin A	*Escherichia coli*	Q47108	III	21.82 kDa	leiomyosarcoma cell line (SKUT-1: <10^5^ AU) fibrosarcoma cell line (HS913T: 10^5^ AU) breast carcinoma cell line (BT549: 10^5^ AU)	[96]
Colicin E1	*Escherichia coli*	P02978	III	57 kDa	fibrosarcoma cell line (HS913T: >10^4^ AU)	[96]
Colicin N	*Escherichia coli*	P08083	III	42 kDa	lung cancer cell lines (H460: >15 µM, H292: >15 µM, H23: >15 µM, and A549: >42 µM) colon cancer cell lines (HCT-116 and HT-29): >42 µM breast cancer cell lines (MDA-MB-231: >42 µM)	[8,97,98]
Pyocin S2	*Pseudomonas aeruginosa* 42A	Q06584	III	73.8 kDa	hepatocellular carcinoma cell line (HepG2: 50 U/mL) multiple myeloma cell line (Im9: 25.5 U/mL) cervical cancer cell line (HeLa S3: At 400 units/mL, cells were inhibited after 2 days of culture) embryonal carcinoma of ovarium cell line (AS-II: At 400 units/mL, cells were inhibited after 4 days of culture)	[99,100]
KL15	*Lactobacillus casei* ATCC 334	KRKLYKWFAHLIKGL	/	1.9 kDa	colon adenocarcinoma cell lines (SW480 and Caco-2): 50 µg/mL or 26.3 µM	[101]
Brevilaterin B	*Brevibacillus laterosporus*	Hmp-Aba-MOIVVKVLKYL-Valinol	/	1.6 kDa	epidermal cancer cell line (A431: 2.75 µg/mL) colon cancer cell line (HCT-29: 1.40 µg/mL) renal cell carcinoma cell line (A-498: 2.33 µg/mL) gastric cancer cell lines (BGC-823: 5.68 µg/mL and SGC-7901: 5.73 µg/mL)	[102]
Brevilaterin C	*Brevibacillus laterosporus*	Hmp-Aba-VOIVVKVLKYL-Valinol	/	1.6 kDa	epidermal cancer cell line (A431: 3.81 µg/mL) colon cancer cell line (HCT-29: 2.21 µg/mL) non-small lung cancer cell line (A549: 4.19 µg/mL) renal cell carcinoma cell line (A-498: 4.16 µg/mL) gastric cancer cell lines (BGC-823: 5.44 µg/mL and SGC-7901: 5.69 µg/mL)	[103]
LHH1	*Lactobacillus casei* HZ1	AFALIAGALYRIFHRR	/	1.9 kDa	colon cancer cell line (HCT-116: 40.83 µM) nasopharyngeal carcinoma cell line (C666-1: 18.27 µM) gastric cancer cell line (MGC803: 31.55 µM)	[11]

HNSCC, head and neck squamous cell carcinoma.

**Table 2:** Completed or ongoing clinical trials on the use of bacteriocins in cancer treatment.

Bacteriocin	Identifier Number	Sponsor	Phase	Status	Cancer
Azurin-p28	NCT00914914	Dr. Tapas K. Das Gupta	I	completed	Solid tumor
Azurin-p28	NCT01975116	Pediatric Brain Tumor Consortium	I	completed	CNS tumor
Nisin ZP	NCT06097468	University of California, San Francisco	I/II	recruiting	OCSCC

CNS, central nervous system; OCSCC, oral cavity squamous cell carcinoma.

## Abbreviations

IARC, International Agency for Research on Cancer; AMPs, antimicrobial peptides; FDA, Food and Drug Administration; DRAMP, Data Repository of Antimicrobial Peptides; Apaf-1, apoptotic protease activating factor-1; ROS, reactive oxygen species; Mcl-1, myeloid cell leukemia-1; c-FLIP, cellular FLICE-like inhibitory protein; CDKs, cyclin-dependent kinases; MDM2, murine double minute 2; ECM, extracellular matrix; EMT, epithelial–mesenchymal transition; MMP, matrix metalloproteinase; VEGF, vascular endothelial growth factor; BBB, blood–brain barrier; His, histidine; Asn, asparagine; OSCC, oral squamous cell carcinoma; DOX, doxorubicin; DMBA, 7,12-dimethylbenz (a) anthracene; 5-FU, 5-fluorouracil; β-CD-NSs, β-cyclodextrin-based nanosponges; NSNE, nisin-shelled nanoemulsions.

## References

[1] Bray F., Laversanne M., Sung H., Ferlay J., Siegel R.L., Soerjomataram I., Jemal A. (2024). Global Cancer Statistics 2022: GLOBOCAN Estimates of Incidence and Mortality Worldwide for 36 Cancers in 185 Countries. CA Cancer J. Clin..

[2] Debela D.T., Muzazu S.G., Heraro K.D., Ndalama M.T., Mesele B.W., Haile D.C., Kitui S.K., Manyazewal T. (2021). New Approaches and Procedures for Cancer Treatment: Current Perspectives. SAGE Open Med..

[3] Naghizadeh S., Mansoori B., Mohammadi A., Sakhinia E., Baradaran B. (2019). Gene Silencing Strategies in Cancer Therapy: An Update for Drug Resistance. Curr. Med. Chem..

[4] Bakare O.O., Gokul A., Wu R., Niekerk L.-A., Klein A., Keyster M. (2021). Biomedical Relevance of Novel Anticancer Peptides in the Sensitive Treatment of Cancer. Biomolecules.

[5] Dong Z., Zhang X., Zhang Q., Tangthianchaichana J., Guo M., Du S., Lu Y. (2024). Anticancer Mechanisms and Potential Anticancer Applications of Antimicrobial Peptides and Their Nano Agents. Int. J. Nanomed..

[6] Kordi M., Borzouyi Z., Chitsaz S., Asmaei M.H., Salami R., Tabarzad M. (2023). Antimicrobial Peptides with Anticancer Activity: Today Status, Trends and Their Computational Design. Arch. Biochem. Biophys..

[7] Papo N., Shai Y. (2005). Host Defense Peptides as New Weapons in Cancer Treatment. Cell. Mol. Life Sci..

[8] Arunmanee W., Ecoy G.A.U., Khine H.E.E., Duangkaew M., Prompetchara E., Chanvorachote P., Chaotham C. (2020). Colicin N Mediates Apoptosis and Suppresses Integrin-Modulated Survival in Human Lung Cancer Cells. Molecules.

[9] Paiva A.D., de Oliveira M.D., de Paula S.O., Baracat-Pereira M.C., Breukink E., Mantovani H.C. (2012). Toxicity of Bovicin HC5 against Mammalian Cell Lines and the Role of Cholesterol in Bacteriocin Activity. Microbiology.

[10] Wang H., Jin J., Pang X., Bian Z., Zhu J., Hao Y., Zhang H., Xie Y. (2022). Plantaricin BM-1 Decreases Viability of SW480 Human Colorectal Cancer Cells by Inducing Caspase-Dependent Apoptosis. Front. Microbiol..

[11] He J.-F., Jin D.-X., Luo X.-G., Zhang T.-C. (2020). LHH1, a Novel Antimicrobial Peptide with Anti-Cancer Cell Activity Identified from Lactobacillus Casei HZ1. AMB Express.

[12] Balcik-Ercin P., Sever B. (2022). An Investigation of Bacteriocin Nisin Anti-Cancer Effects and FZD7 Protein Interactions in Liver Cancer Cells. Chem. Biol. Interact..

[13] Cornut G., Fortin C., Soulières D. (2008). Antineoplastic Properties of Bacteriocins: Revisiting Potential Active Agents. Am. J. Clin. Oncol..

[14] Kaur S., Kaur S. (2015). Bacteriocins as Potential Anticancer Agents. Front. Pharmacol..

[15] Garcia-Gutierrez E., Mayer M.J., Cotter P.D., Narbad A. (2018). Gut Microbiota as a Source of Novel Antimicrobials. Gut Microbes.

[16] Darbandi A., Asadi A., Mahdizade Ari M., Ohadi E., Talebi M., Halaj Zadeh M., Darb Emamie A., Ghanavati R., Kakanj M. (2021). Bacteriocins: Properties and Potential Use as Antimicrobials. J. Clin. Lab. Anal..

[17] Daba G.M., Elkhateeb W.A. (2024). Ribosomally Synthesized Bacteriocins of Lactic Acid Bacteria: Simplicity yet Having Wide Potentials—A Review. Int. J. Biol. Macromol..

[18] Baindara P., Korpole S., Grover V. (2018). Bacteriocins: Perspective for the Development of Novel Anticancer Drugs. Appl. Microbiol. Biotechnol..

[19] Anjana A., Tiwari S.K. (2022). Bacteriocin-Producing Probiotic Lactic Acid Bacteria in Controlling Dysbiosis of the Gut Microbiota. Front. Cell. Infect. Microbiol..

[20] Nes I.F., Diep D.B., Håvarstein L.S., Brurberg M.B., Eijsink V., Holo H. (1996). Biosynthesis of Bacteriocins in Lactic Acid Bacteria. Antonie Van Leeuwenhoek.

[21] Klaenhammer T.R. (1993). Genetics of Bacteriocins Produced by Lactic Acid Bacteria. FEMS Microbiol. Rev..

[22] Wu A., Fu Y., Kong L., Shen Q., Liu M., Zeng X., Wu Z., Guo Y., Pan D. (2021). Production of a Class IIb Bacteriocin with Broad-Spectrum Antimicrobial Activity in Lactiplantibacillus Plantarum RUB1. Probiotics Antimicrob. Proteins.

[23] Dubey S., Diep D.B., Evensen Ø., Munang’andu H.M. (2022). Garvicin KS, a Broad-Spectrum Bacteriocin Protects Zebrafish Larvae against Lactococcus Garvieae Infection. Int. J. Mol. Sci..

[24] Juturu V., Wu J.C. (2018). Microbial Production of Bacteriocins: Latest Research Development and Applications. Biotechnol. Adv..

[25] Cui Y., Luo L., Wang X., Lu Y., Yi Y., Shan Y., Liu B., Zhou Y., Lü X. (2021). Mining, Heterologous Expression, Purification, Antibactericidal Mechanism, and Application of Bacteriocins: A Review. Compr. Rev. Food Sci. Food Saf..

[26] Lin T.-Y., Weibel D.B. (2016). Organization and Function of Anionic Phospholipids in Bacteria. Appl. Microbiol. Biotechnol..

[27] Zhang Q.-Y., Yan Z.-B., Meng Y.-M., Hong X.-Y., Shao G., Ma J.-J., Cheng X.-R., Liu J., Kang J., Fu C.-Y. (2021). Antimicrobial Peptides: Mechanism of Action, Activity and Clinical Potential. Mil. Med. Res..

[28] Molujin A.M., Abbasiliasi S., Nurdin A., Lee P.-C., Gansau J.A., Jawan R. (2022). Bacteriocins as Potential Therapeutic Approaches in the Treatment of Various Cancers: A Review of In Vitro Studies. Cancers.

[29] Magana M., Pushpanathan M., Santos A.L., Leanse L., Fernandez M., Ioannidis A., Giulianotti M.A., Apidianakis Y., Bradfute S., Ferguson A.L. (2020). The Value of Antimicrobial Peptides in the Age of Resistance. Lancet Infect. Dis..

[30] Reuben R.C., Torres C. (2024). Bacteriocins: Potentials and Prospects in Health and Agrifood Systems. Arch. Microbiol..

[31] Radaic A., de Jesus M.B., Kapila Y.L. (2020). Bacterial Anti-Microbial Peptides and Nano-Sized Drug Delivery Systems: The State of the Art toward Improved Bacteriocins. J. Control. Release.

[32] Shi G., Kang X., Dong F., Liu Y., Zhu N., Hu Y., Xu H., Lao X., Zheng H. (2022). DRAMP 3.0: An Enhanced Comprehensive Data Repository of Antimicrobial Peptides. Nucleic Acids Res..

[33] Zacharof M.P., Lovitt R.W. (2012). Bacteriocins Produced by Lactic Acid Bacteria a Review Article. APCBEE Procedia.

[34] Cotter P.D., Hill C., Ross R.P. (2005). Bacteriocins: Developing Innate Immunity for Food. Nat. Rev. Microbiol..

[35] Sahl H.G., Bierbaum G. (1998). Lantibiotics: Biosynthesis and Biological Activities of Uniquely Modified Peptides from Gram-Positive Bacteria. Annu. Rev. Microbiol..

[36] And H.C., Hoover D.G. (2003). Bacteriocins and Their Food Applications. Compr. Rev. Food Sci. Food Saf..

[37] Riedl S., Rinner B., Asslaber M., Schaider H., Walzer S., Novak A., Lohner K., Zweytick D. (2011). In Search of a Novel Target—Phosphatidylserine Exposed by Non-Apoptotic Tumor Cells and Metastases of Malignancies with Poor Treatment Efficacy. Biochim. Biophys. Acta (BBA)—Biomembr..

[38] Kufe D.W. (2009). Mucins in Cancer: Function, Prognosis and Therapy. Nat. Rev. Cancer.

[39] Groux-Degroote S., Guérardel Y., Delannoy P. (2017). Gangliosides: Structures, Biosynthesis, Analysis, and Roles in Cancer. ChemBioChem.

[40] Vicente C.M., da Silva D.A., Sartorio P.V., Silva T.D., Saad S.S., Nader H.B., Forones N.M., Toma L. (2018). Heparan Sulfate Proteoglycans in Human Colorectal Cancer. Anal. Cell. Pathol..

[41] Zalba S., ten Hagen T.L.M. (2017). Cell Membrane Modulation as Adjuvant in Cancer Therapy. Cancer Treat. Rev..

[42] Signati L., Allevi R., Piccotti F., Albasini S., Villani L., Sevieri M., Bonizzi A., Corsi F., Mazzucchelli S. (2021). Ultrastructural Analysis of Breast Cancer Patient-Derived Organoids. Cancer Cell Int..

[43] Zeisig R., Koklic T., Wiesner B., Fichtner I., Sentjurc M. (2007). Increase in Fluidity in the Membrane of MT3 Breast Cancer Cells Correlates with Enhanced Cell Adhesion in Vitro and Increased Lung Metastasis in NOD/SCID Mice. Arch. Biochem. Biophys..

[44] Ren J., Hamada J., Okada F., Takeichi N., Morikawa K., Hosokawa M., Kobayashi H. (1990). Correlation between the Presence of Microvilli and the Growth or Metastatic Potential of Tumor Cells. Jpn. J. Cancer Res..

[45] Aghamiri S., Zandsalimi F., Raee P., Abdollahifar M.-A., Tan S.C., Low T.Y., Najafi S., Ashrafizadeh M., Zarrabi A., Ghanbarian H. (2021). Antimicrobial Peptides as Potential Therapeutics for Breast Cancer. Pharmacol. Res..

[46] Chan S.C., Hui L., Chen H.M. (1998). Enhancement of the Cytolytic Effect of Anti-Bacterial Cecropin by the Microvilli of Cancer Cells. Anticancer. Res..

[47] Moll G.N., Konings W.N., Driessen A.J. (1999). Bacteriocins: Mechanism of Membrane Insertion and Pore Formation. Antonie Van Leeuwenhoek.

[48] Yoneyama F., Imura Y., Ohno K., Zendo T., Nakayama J., Matsuzaki K., Sonomoto K. (2009). Peptide-Lipid Huge Toroidal Pore, a New Antimicrobial Mechanism Mediated by a Lactococcal Bacteriocin, Lacticin Q. Antimicrob. Agents Chemother..

[49] Tahara T., Kanatani K. (1996). Isolation, Partial Characterization and Mode of Action of Acidocin J1229, a Bacteriocin Produced by Lactobacillus Acidophilus JCM 1229. J. Appl. Bacteriol..

[50] Tahara T., Oshimura M., Umezawa C., Kanatani K. (1996). Isolation, Partial Characterization, and Mode of Action of Acidocin J1132, a Two-Component Bacteriocin Produced by Lactobacillus Acidophilus JCM 1132. Appl. Environ. Microbiol..

[51] Yeaman M.R., Yount N.Y. (2003). Mechanisms of Antimicrobial Peptide Action and Resistance. Pharmacol. Rev..

[52] Tripathi A.K., Vishwanatha J.K. (2022). Role of Anti-Cancer Peptides as Immunomodulatory Agents: Potential and Design Strategy. Pharmaceutics.

[53] Lopes J.L.S., Gómara M.J., Haro I., Tonarelli G., Beltramini L.M. (2013). Contribution of the Tyr-1 in Plantaricin149a to Disrupt Phospholipid Model Membranes. Int. J. Mol. Sci..

[54] Li M., Yoneyama F., Toshimitsu N., Zendo T., Nakayama J., Sonomoto K. (2013). Lethal Hydroxyl Radical Accumulation by a Lactococcal Bacteriocin, Lacticin Q. Antimicrob. Agents Chemother..

[55] Riedlová K., Dolejšová T., Fišer R., Cwiklik L. (2022). H1 Helix of Colicin U Causes Phospholipid Membrane Permeation. Biochim. Biophys. Acta Biomembr..

[56] Sobko A.A., Kotova E.A., Antonenko Y.N., Zakharov S.D., Cramer W.A. (2006). Lipid Dependence of the Channel Properties of a Colicin E1-Lipid Toroidal Pore. J. Biol. Chem..

[57] Driessen A.J., van den Hooven H.W., Kuiper W., van de Kamp M., Sahl H.G., Konings R.N., Konings W.N. (1995). Mechanistic Studies of Lantibiotic-Induced Permeabilization of Phospholipid Vesicles. Biochemistry.

[58] Moll G.N., Roberts G.C., Konings W.N., Driessen A.J. (1996). Mechanism of Lantibiotic-Induced Pore-Formation. Antonie Van Leeuwenhoek.

[59] Zhu L., Zeng J., Wang C., Wang J. (2022). Structural Basis of Pore Formation in the Mannose Phosphotransferase System by Pediocin PA-1. Appl. Environ. Microbiol..

[60] Mårtensson C.U., Doan K.N., Becker T. (2017). Effects of Lipids on Mitochondrial Functions. Biochim. Biophys. Acta Mol. Cell Biol. Lipids.

[61] Joo N.E., Ritchie K., Kamarajan P., Miao D., Kapila Y.L. (2012). Nisin, an Apoptogenic Bacteriocin and Food Preservative, Attenuates HNSCC Tumorigenesis via CHAC1. Cancer Med..

[62] Hetz C., Bono M.R., Barros L.F., Lagos R. (2002). Microcin E492, a Channel-Forming Bacteriocin from Klebsiella Pneumoniae, Induces Apoptosis in Some Human Cell Lines. Proc. Natl. Acad. Sci. USA.

[63] Punj V., Bhattacharyya S., Saint-Dic D., Vasu C., Cunningham E.A., Graves J., Yamada T., Constantinou A.I., Christov K., White B. (2004). Bacterial Cupredoxin Azurin as an Inducer of Apoptosis and Regression in Human Breast Cancer. Oncogene.

[64] Montalto F.I., De Amicis F. (2020). Cyclin D1 in Cancer: A Molecular Connection for Cell Cycle Control, Adhesion and Invasion in Tumor and Stroma. Cells.

[65] Hosseini S.S., Goudarzi H., Ghalavand Z., Hajikhani B., Rafeieiatani Z., Hakemi-Vala M. (2020). Anti-Proliferative Effects of Cell Wall, Cytoplasmic Extract of Lactococcus Lactis and Nisin through down-Regulation of Cyclin D1 on SW480 Colorectal Cancer Cell Line. Iran. J. Microbiol..

[66] Engeland K. (2018). Cell Cycle Arrest through Indirect Transcriptional Repression by P53: I Have a DREAM. Cell Death Differ..

[67] Yamada T., Goto M., Punj V., Zaborina O., Chen M.L., Kimbara K., Majumdar D., Cunningham E., Das Gupta T.K., Chakrabarty A.M. (2002). Bacterial Redox Protein Azurin, Tumor Suppressor Protein P53, and Regression of Cancer. Proc. Natl. Acad. Sci. USA.

[68] Yamada T., Mehta R.R., Lekmine F., Christov K., King M.L., Majumdar D., Shilkaitis A., Green A., Bratescu L., Beattie C.W. (2009). A Peptide Fragment of Azurin Induces a P53-Mediated Cell Cycle Arrest in Human Breast Cancer Cells. Mol. Cancer Ther..

[69] Norouzi Z., Salimi A., Halabian R., Fahimi H. (2018). Nisin, a Potent Bacteriocin and Anti-Bacterial Peptide, Attenuates Expression of Metastatic Genes in Colorectal Cancer Cell Lines. Microb. Pathog..

[70] Das S., Amin S.A., Jha T. (2021). Inhibitors of Gelatinases (MMP-2 and MMP-9) for the Management of Hematological Malignancies. Eur. J. Med. Chem..

[71] El-Sayed Ibrahim N., Morsy H., Abdelgwad M. (2021). The Comparative Effect of Nisin and Thioridazine as Potential Anticancer Agents on Hepatocellular Carcinoma. Rep. Biochem. Mol. Biol..

[72] Maher S., McClean S. (2006). Investigation of the Cytotoxicity of Eukaryotic and Prokaryotic Antimicrobial Peptides in Intestinal Epithelial Cells in Vitro. Biochem. Pharmacol..

[73] Begde D., Bundale S., Mashitha P., Rudra J., Nashikkar N., Upadhyay A. (2011). Immunomodulatory Efficacy of Nisin—A Bacterial Lantibiotic Peptide. J. Pept. Sci..

[74] Prince A., Tiwari A., Ror P., Sandhu P., Roy J., Jha S., Mallick B., Akhter Y., Saleem M. (2019). Attenuation of Neuroblastoma Cell Growth by Nisin Is Mediated by Modulation of Phase Behavior and Enhanced Cell Membrane Fluidity. Phys. Chem. Chem. Phys..

[75] Sadri H., Aghaei M., Akbari V. (2022). Nisin Induces Apoptosis in Cervical Cancer Cells via Reactive Oxygen Species Generation and Mitochondrial Membrane Potential Changes. Biochem. Cell Biol..

[76] Zainodini N., Hassanshahi G., Hajizadeh M., Khanamani Falahati-Pour S., Mahmoodi M., Mirzaei M.R. (2018). Nisin Induces Cytotoxicity and Apoptosis in Human Asterocytoma Cell Line (SW1088). Asian Pac. J. Cancer Prev..

[77] Kamarajan P., Hayami T., Matte B., Liu Y., Danciu T., Ramamoorthy A., Worden F., Kapila S., Kapila Y. (2015). Nisin ZP, a Bacteriocin and Food Preservative, Inhibits Head and Neck Cancer Tumorigenesis and Prolongs Survival. PLoS ONE.

[78] Kamarajan P., Ateia I., Shin J.M., Fenno J.C., Le C., Zhan L., Chang A., Darveau R., Kapila Y.L. (2020). Periodontal Pathogens Promote Cancer Aggressivity via TLR/MyD88 Triggered Activation of Integrin/FAK Signaling That Is Therapeutically Reversible by a Probiotic Bacteriocin. PLoS Pathog..

[79] Lewies A., Wentzel J.F., Miller H.C., Du Plessis L.H. (2018). The Antimicrobial Peptide Nisin Z Induces Selective Toxicity and Apoptotic Cell Death in Cultured Melanoma Cells. Biochimie.

[80] Patil S.M., Kunda N.K. (2022). Nisin ZP, an Antimicrobial Peptide, Induces Cell Death and Inhibits Non-Small Cell Lung Cancer (NSCLC) Progression in Vitro in 2D and 3D Cell Culture. Pharm. Res..

[81] Lee D.G., Hahm K.-S., Park Y., Kim H.-Y., Lee W., Lim S.-C., Seo Y.-K., Choi C.-H. (2005). Functional and Structural Characteristics of Anticancer Peptide Pep27 Analogues. Cancer Cell Int..

[82] Beaulieu L. (2004). Production, Purification et Caracterisation de La Pediocine PA-1 Naturelle et de Ses Formes Recombinantes: Contribution a La Mise En Evidence d’une Nouvelle Activite Biologique. Ph.D. Thesis.

[83] Villarante K.I., Elegado F.B., Iwatani S., Zendo T., Sonomoto K., de Guzman E.E. (2011). Purification, Characterization and in Vitro Cytotoxicity of the Bacteriocin from Pediococcus Acidilactici K2a2-3 against Human Colon Adenocarcinoma (HT29) and Human Cervical Carcinoma (HeLa) Cells. World J. Microbiol. Biotechnol..

[84] Kumar B. (2012). In Vitro Cytotoxicity of Native and Rec-Pediocin CP2 Against Cancer Cell Lines: A Comparative Study. Pharm. Anal. Acta.

[85] De Giani A., Bovio F., Forcella M., Fusi P., Sello G., Di Gennaro P. (2019). Identification of a Bacteriocin-like Compound from Lactobacillus Plantarum with Antimicrobial Activity and Effects on Normal and Cancerogenic Human Intestinal Cells. AMB Express.

[86] Zhao H., Sood R., Jutila A., Bose S., Fimland G., Nissen-Meyer J., Kinnunen P.K.J. (2006). Interaction of the Antimicrobial Peptide Pheromone Plantaricin A with Model Membranes: Implications for a Novel Mechanism of Action. Biochim. Biophys. Acta.

[87] Sand S.L., Oppegård C., Ohara S., Iijima T., Naderi S., Blomhoff H.K., Nissen-Meyer J., Sand O. (2010). Plantaricin A, a Peptide Pheromone Produced by Lactobacillus Plantarum, Permeabilizes the Cell Membrane of Both Normal and Cancerous Lymphocytes and Neuronal Cells. Peptides.

[88] Baindara P., Gautam A., Raghava G.P.S., Korpole S. (2017). Anticancer Properties of a Defensin like Class IId Bacteriocin Laterosporulin10. Sci. Rep..

[89] Ankaiah D., Esakkiraj P., Perumal V., Ayyanna R., Venkatesan A. (2017). Probiotic Characterization of *Enterococcus faecium* Por1: Cloning, over Expression of Enterocin-A and Evaluation of Antibacterial, Anti-Cancer Properties. J. Funct. Foods.

[90] Ankaiah D., Palanichamy E., Antonyraj C.B., Ayyanna R., Perumal V., Ahamed S.I.B., Arul V. (2018). Cloning, Overexpression, Purification of Bacteriocin Enterocin-B and Structural Analysis, Interaction Determination of Enterocin-A, B against Pathogenic Bacteria and Human Cancer Cells. Int. J. Biol. Macromol..

[91] Varas M.A., Muñoz-Montecinos C., Kallens V., Simon V., Allende M.L., Marcoleta A.E., Lagos R. (2020). Exploiting Zebrafish Xenografts for Testing the in Vivo Antitumorigenic Activity of Microcin E492 Against Human Colorectal Cancer Cells. Front. Microbiol..

[92] Yang D.-S., Miao X.-D., Ye Z.-M., Feng J., Xu R.-Z., Huang X., Ge F.-F. (2005). Bacterial Redox Protein Azurin Induce Apoptosis in Human Osteosarcoma U2OS Cells. Pharmacol. Res..

[93] Kwan J.M., Fialho A.M., Kundu M., Thomas J., Hong C.S., Das Gupta T.K., Chakrabarty A.M. (2009). Bacterial Proteins as Potential Drugs in the Treatment of Leukemia. Leuk. Res..

[94] Mohamed M., Mostafa S. (2010). Azurin as Antitumor Protein and Its Effect on the Cancer Cell Lines. Curr. Res. J. Biol. Sci..

[95] Cho J.-H., Lee M.-H., Cho Y.-J., Park B.-S., Kim S., Kim G.-C. (2011). The Bacterial Protein Azurin Enhances Sensitivity of Oral Squamous Carcinoma Cells to Anticancer Drugs. Yonsei Med. J..

[96] Chumchalová J., Smarda J. (2003). Human Tumor Cells Are Selectively Inhibited by Colicins. Folia Microbiol..

[97] Arunmanee W., Duangkaew M., Taweecheep P., Aphicho K., Lerdvorasap P., Pitchayakorn J., Intasuk C., Jiraratmetacon R., Syamsidi A., Chanvorachote P. (2021). Resurfacing Receptor Binding Domain of Colicin N to Enhance Its Cytotoxic Effect on Human Lung Cancer Cells. Comput. Struct. Biotechnol. J..

[98] Duangkaew M., Arunmanee W. (2022). In Vitro Screening for Cytotoxic Effect of Pore Forming Colicin N and Its Domains on Human Cancer Cells. Trop. Life Sci. Res..

[99] Abdi-Ali A., Worobec E.A., Deezagi A., Malekzadeh F. (2004). Cytotoxic Effects of Pyocin S2 Produced by Pseudomonas Aeruginosa on the Growth of Three Human Cell Lines. Can. J. Microbiol..

[100] Watanabe T., Saito H. (1980). Cytotoxicity of Pyocin S2 to Tumor and Normal Cells and Its Interaction with Cell Surfaces. Biochim. Biophys. Acta.

[101] Chen Y.-C., Tsai T.-L., Ye X.-H., Lin T.-H. (2015). Anti-Proliferative Effect on a Colon Adenocarcinoma Cell Line Exerted by a Membrane Disrupting Antimicrobial Peptide KL15. Cancer Biol. Ther..

[102] Chen Z., Wang L., Liu Y., Han P., Hong D., Li S., Ma A., Jia Y. (2022). Brevilaterin B from Brevibacillus Laterosporus Has Selective Antitumor Activity and Induces Apoptosis in Epidermal Cancer. World J. Microbiol. Biotechnol..

[103] Chen Z., Wang L., Hong D., Liu Y., Han P., Li S., Jia Y. (2022). Broad-Spectrum Cytotoxicity to Cancer Cells of Brevilaterin C from Brevibacillus Laterosporus and Its Specific Mechanism on Human Epidermal Cancer Cells. J. Cell. Biochem..

[104] Yaghoubi A., Khazaei M., Avan A., Hasanian S.M., Cho W.C., Soleimanpour S. (2020). P28 Bacterial Peptide, as an Anticancer Agent. Front. Oncol..

[105] Warso M.A., Richards J.M., Mehta D., Christov K., Schaeffer C., Rae Bressler L., Yamada T., Majumdar D., Kennedy S.A., Beattie C.W. (2013). A First-in-Class, First-in-Human, Phase I Trial of P28, a Non-HDM2-Mediated Peptide Inhibitor of P53 Ubiquitination in Patients with Advanced Solid Tumours. Br. J. Cancer.

[106] Lulla R.R., Goldman S., Yamada T., Beattie C.W., Bressler L., Pacini M., Pollack I.F., Fisher P.G., Packer R.J., Dunkel I.J. (2016). Phase I Trial of P28 (NSC745104), a Non-HDM2-Mediated Peptide Inhibitor of P53 Ubiquitination in Pediatric Patients with Recurrent or Progressive Central Nervous System Tumors: A Pediatric Brain Tumor Consortium Study. Neuro Oncol..

[107] Chauhan S., Dhawan D.K., Saini A., Preet S. (2021). Antimicrobial Peptides against Colorectal Cancer-a Focused Review. Pharmacol. Res..

[108] Preet S., Bharati S., Panjeta A., Tewari R., Rishi P. (2015). Effect of Nisin and Doxorubicin on DMBA-Induced Skin Carcinogenesis--a Possible Adjunct Therapy. Tumour Biol..

[109] Mohammadi P., Zangeneh M., Mohammadi-Motlagh H.-R., Khademi F. (2020). The Antimicrobial Peptide, Nisin, Synergistically Enhances the Cytotoxic and Apoptotic Effects of Rituximab Treatment on Human Burkitt’s Lymphoma Cell Lines. Rep. Biochem. Mol. Biol..

[110] Baxter A.A., Lay F.T., Poon I.K.H., Kvansakul M., Hulett M.D. (2017). Tumor Cell Membrane-Targeting Cationic Antimicrobial Peptides: Novel Insights into Mechanisms of Action and Therapeutic Prospects. Cell. Mol. Life Sci..

[111] Fathizadeh H., Saffari M., Esmaeili D., Moniri R., Kafil H.S. (2021). Bacteriocins: New Potential Therapeutic Candidates in Cancer Therapy. Curr. Mol. Med..

[112] Bengtsson T., Selegård R., Musa A., Hultenby K., Utterström J., Sivlér P., Skog M., Nayeri F., Hellmark B., Söderquist B. (2020). Plantaricin NC8 Aβ Exerts Potent Antimicrobial Activity against Staphylococcus Spp. and Enhances the Effects of Antibiotics. Sci. Rep..

[113] Oppegård C., Rogne P., Kristiansen P.E., Nissen-Meyer J. (2010). Structure Analysis of the Two-Peptide Bacteriocin Lactococcin G by Introducing D-Amino Acid Residues. Microbiology.

[114] Sánchez-Hidalgo M., Montalbán-López M., Cebrián R., Valdivia E., Martínez-Bueno M., Maqueda M. (2011). AS-48 Bacteriocin: Close to Perfection. Cell. Mol. Life Sci..

[115] O’Shea E.F., O’Connor P.M., Cotter P.D., Ross R.P., Hill C. (2010). Synthesis of Trypsin-Resistant Variants of the Listeria-Active Bacteriocin Salivaricin P. Appl. Environ. Microbiol..

[116] Fathizadeh H., Saffari M., Esmaeili D., Moniri R., Mahabadi J.A. (2021). Anticancer Effect of Enterocin A-Colicin E1 Fusion Peptide on the Gastric Cancer Cell. Probiotics Antimicrob. Proteins.

[117] Haider T., Pandey V., Behera C., Kumar P., Gupta P.N., Soni V. (2022). Nisin and Nisin-Loaded Nanoparticles: A Cytotoxicity Investigation. Drug Dev. Ind. Pharm..

[118] Khazaei Monfared Y., Mahmoudian M., Cecone C., Caldera F., Zakeri-Milani P., Matencio A., Trotta F. (2022). Stabilization and Anticancer Enhancing Activity of the Peptide Nisin by Cyclodextrin-Based Nanosponges against Colon and Breast Cancer Cells. Polymers.

[119] Hashad R.A., Singla R., Kaur Bhangu S., Jap E., Zhu H., Peleg A.Y., Blakeway L., Hagemeyer C.E., Cavalieri F., Ashokkumar M. (2022). Chemoenzymatic Surface Decoration of Nisin-Shelled Nanoemulsions: Novel Targeted Drug-Nanocarriers for Cancer Applications. Ultrason. Sonochem..

